# The impact of Joint Commission International accreditation on time periods in the operating room: A retrospective observational study

**DOI:** 10.1371/journal.pone.0204301

**Published:** 2018-09-21

**Authors:** Takenori Inomata, Ju Mizuno, Masao Iwagami, Shiori Kawasaki, Akie Shimada, Eiichi Inada, Tina Shiang, Atsushi Amano

**Affiliations:** 1 Juntendo University Faculty of Medicine, Department of Strategic Operating Room Management and Improvement, Tokyo, Japan; 2 Juntendo University Faculty of Medicine, Department of Ophthalmology, Tokyo, Japan; 3 Juntendo University Faculty of Medicine, Department of Anesthesia, Tokyo, Japan; 4 London School of Hygiene and Tropical Medicine, Department of Non-Communicable Disease Epidemiology; 5 Juntendo University Faculty of Medicine, Department of Cardiovascular Surgery, Tokyo, Japan; 6 University of Massachusetts Medical School, Department of Radiology, Massachusetts, MA, United States of America; Jinling Clinical Medical College of Nanjing Medical University, CHINA

## Abstract

The Joint Commission International (JCI) is responsible for upholding standards in healthcare and organizations in compliance receive accreditation. JCI requires quality improvement on patient safety goals, but requirements may prolong the total procedure/surgery time and reduce efficiency. Here, we evaluate the impact of JCI requirements on time periods in the operating room. We included patients who received elective and emergency surgeries under general anesthesia at Juntendo University Hospital between December 2014 and June 2016. Patients were classified as before and after JCI accreditation on December 12, 2015. The primary outcome was total procedure/surgery time. Secondary outcomes include five time periods comprising the total procedure/surgery time: pre-anesthesia time, anesthesia induction time, procedure/surgery time, anesthesia awareness time and post-anesthesia time. We compared these time periods between patients before and after JCI accreditation and patients were matched for age, sex and the specific type of surgery. Although total procedure/surgery time did not change significantly, pre-anesthesia time significantly increased (8.2 ± 6.9 minutes vs. 8.5 ± 6.9 minutes, before vs. after JCI, respectively, p = 0.028) and anesthesia induction time significantly decreased (34.4 ± 16.1 minutes vs. 33.6 ± 15.4 minutes, before vs. after JCI, respectively, p = 0.037) after JCI accreditation. Other secondary study outcomes did not change significantly. Quality improvement initiatives associated with time periods in the operating room can be achieved without undermining efficiency.

## Introduction

World population aging has resulted in a significant growth in demand for surgical services [1]. Japan faces the challenges of a rapidly aging society and changes in the universal medical insurance system, and healthcare providers must consequently adapt their management and marketing strategies.

Juntendo University Hospital (JUH), founded in 1838, is one of Japan’s oldest private medical schools in central Tokyo and receives 3,894 outpatients and 941 inpatients per day. There are more than 10,000 surgical cases at JUH per year, resulting in an overuse of surgical services and many add-on surgeries in an attempt to compensate the steady increase in surgical volume. The number of add-on surgeries are expected to decrease with the completion of the new operating rooms in March 2014, but it is necessary to further optimize operating room efficiency [2]. These improvements will involve a delicate balance between patient safety and hospital efficiency [3].

The Joint Commission International (JCI) is responsible for upholding patient safety and accrediting healthcare organizations in compliance with standards. JUH was accredited on December 12, 2015. JCI requires quality improvement for international patient safety goals (IPSG) defining important issues concerning patient safety. IPSG helps confirm correct patient identification, encourages effective communication between patients and medical staffs, improves the safety of high-alert medication administration, and ensures safe surgeries (correct surgical site, procedures, and patient for the surgery) [4]. JCI accreditation is expected to improve patient safety associated with surgical operations, but there is concern that these changes will prolong total procedure/surgery time (TPT) in the operating room and reduce efficiency. To date, there has been no study examining the impact of ISPG procedures on operating room efficiency.

Hospitals need to make effective use of human resources because of the sharp decrease in active medical personnel and overall decrease in the Japanese workforce [5]. Since fixed costs such as payments to nurses and anesthesiologists cannot be changed, it is imperative that we improve operating room efficiency. In this study, we aim to examine the impact of JCI accreditation on operating room efficiency by comparing relevant time periods in the operating room for patients who received surgeries before and after JCI accreditation at JUH.

## Materials and methods

### Study design

Retrospective observational study. A requirement for a written informed consent was waived due to the retrospective observational nature of the study, and it was carried out using the opt-out method on our hospital website. All data were fully anonymized before we accessed them. The study was approved by the Institutional Review Board and the Medical Ethics Committee of JUH (16–153). This project was conducted in adherence with the tenets of the Declaration of Helsinki.

### Accreditation by Joint Commission International

JUH became JCI accredited on December 12, 2015. Every three years, inspectors visit hospitals to observe hospital operations, conducts interviews, and review medical documentation, and hospitals that meet compliance standards set forth by the JCI receive accreditation. The goal is to evaluate care, standardize hospital processes and provide education and promote quality improvement for the organizations under survey.

### Participants

All patients having elective and emergency surgeries under general anesthesia in JUH between December 2014 and June 2016 were considered in this retrospective study. Medical records were reviewed and patient demographics (age and sex), date of surgery, surgical department, specific type of surgery and the time periods in the operating room were collected. Patients were divided into before and after JCI accreditation groups.

### Outcome measures

**Fig 1:**
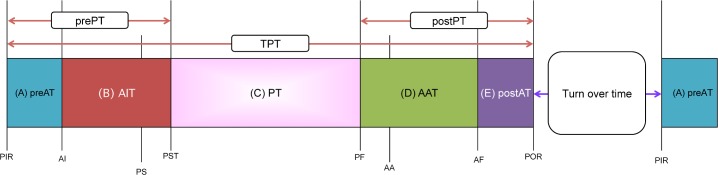
The glossary of time periods in the operating room. **The glossary of time periods was divided into five time intervals.** (A). preAT = pre-anesthesia time, (B). AIT = anesthesia induction time, (C). PT = procedure/surgery time, (D). AAT = anesthesia awareness time, (E). postAT = post-anesthesia time, prePT = pre-procedure/surgery time (A+B), postPT = post-procedure/surgery time (C+D), TPT = total procedure/surgery time (prePT+C+postPT), PIR = patient in room, AI = anesthesia induction, PS = position/prep start, PST = procedure/surgery start time, PF = procedure/surgery finish time, AA = anesthesia awareness, AF = anesthesia finish time, POR = patient out of room.

In this study, we divided TPT into five more specific time periods (Fig 1) according to previous studies [2, 6–8]. Time periods were defined as pre-anesthesia time (preAT), anesthesia induction time (AIT), procedure/surgery time (PT), anesthesia awareness time (AAT) and post-anesthesia time (postAT). As shown in Fig 1, preAT is defined as the time elapsed in minutes between when the patient enters the operating room (patient in room, PIR) and anesthesia induction (the time at which the patient inhales oxygen from an anesthetic machine, AI), including the attachment of monitors such as an electrocardiogram and blood pressure gauge and sign in (S1 Table). AIT is defined as the time elapsed in minutes between the start of anesthesia induction and the start of surgery (procedure/surgery start time, PST) indicated by the time out. PT is defined as the time elapsed in minutes between the start and finish of surgery (the procedure/surgery start time to the procedure/surgery finish time, PST, PF, respectively). AAT is defined as the time elapsed in minutes between PF to the time the patient’s oxygen is discontinued from the anesthetic machine (anesthesia finish time, AF). postAT is defined as the time elapsed in minutes between the AF and the time the patient exits the room (patient out of room, POR). Then, the pre-procedure/surgery time (prePT) was calculated from the sum of preAT and AIT. The post-procedure/surgery time (postPT) was calculated from the sum of AAT and postAT. The TPT was calculated from the sum of preAT to postAT.

### Analysis

Patient characteristics were compared between those who had surgery before and after JCI accreditation using the chi-square test for age group, sex and specific type of surgery. To fairly compare patient outcomes before and after JCI accreditation, we matched patients for age (every 10-year category) and sex (Table 1), and specific type of surgery (Table 2). Matching on specific type of surgery was necessary in the current study because the distribution of surgeries was significantly different among patients and operation time largely depends on the type of surgery (S2 and S3 Tables). We selected matched patients randomly from both groups on a 1:1 basis. We compared the time periods in the operating room between the matched patients before and after JCI accreditation using the paired t-test.

**Table 1:** Characteristics of patients before and after matching for age, sex and specific type of surgery.

	Before matching			After matching		
	Before JCI	After JCI	p value	Before JCI	After JCI	p value
	N = 8,835	N = 4,453		N = 3,222	N = 3,222	
	n (%)	n (%)		n (%)	n (%)	
Age (y.o.)			0.679			1.000
0–9	1,612 (18.3)	785 (17.6)		573 (17.8)	573 (17.8)	
10–19	401 (4.5)	231 (5.2)		114 (3.5)	114 (3.5)	
20–29	429 (4.9)	230 (5.2)		129 (4.0)	129 (4.0)	
30–39	987 (11.2)	480 (10.8)		384 (11.9)	384 (11.9)	
40–49	1,144 (13.0)	569 (12.8)		444 (13.8)	444 (13.8)	
50–59	892 (10.1)	469 (10.5)		315 (9.8)	315 (9.8)	
60–69	1,440 (16.3)	724 (16.3)		542 (16.8)	542 (16.8)	
70–79	1,455(16.5)	706 (15.9)		555 (17.2)	555 (17.2)	
80–89	456 (5.2)	248 (5.6)		163 (5.1)	163 (5.1)	
90–99	19 (0.2)	11 (0.3)		3 (0.1)	3 (0.1)	
Sex						
Male	4,199 (47.5)	2151 (48.3)	0.397	1,505 (46.7)	1,505 (46.7)	1.000
Female	4,636 (52.5)	2302 (51.7)		1,717 (53.3)	1,717 (53.3)	

JCI; Joint Commission International, p value is calculated by chi-square test.

**Table 2:** The distribution of the department after matching for age, sex and specific type of surgery.

Department	Before JCI	After JCI	p value
Breast oncology	184	183	0.998
Cardiovascular surgery	233	233	
Coloproctological surgery	119	117	
Esophageal and Gastroenterological surgery	76	77	
Gynecology	510	485	
Hepatobiliary-Pancreatic surgery	116	118	
Neurosurgery	199	198	
Obstetrics	164	190	
Ophthalmology	114	113	
Orthopedics	240	247	
Otolaryngology	224	224	
Pediatric surgery	414	412	
Plastic surgery	120	126	
Thoracic surgery	206	205	
Urology	277	275	
Others	26	19	
Total	3,222	3,222	

JCI; Joint Commission International, p value is calculated by chi-square test.

Next, we conducted subgroup analyses by focusing on three common and standardized surgeries: total hip arthroplasty, total knee arthroplasty, and laparoscopic cholecystectomy. We compared the time periods in the operating room using the unpaired t-test between patients before and after JCI accreditation in each surgical group. All data were analyzed with STATA version 14 (Stata Corp, Texas, US).

## Results

### Characteristics of patients

A total of 13,288 patients (median age 44.9, [interquartile range 25–68], male 47.5%) received surgical treatments under general anesthesia at JUH during the study period. Although the age-sex distribution was similar (Table 1), the distribution of surgeries among different surgical departments before and after JCI accreditation was significantly different (S2 Table).

### Main results

**Fig 2:**
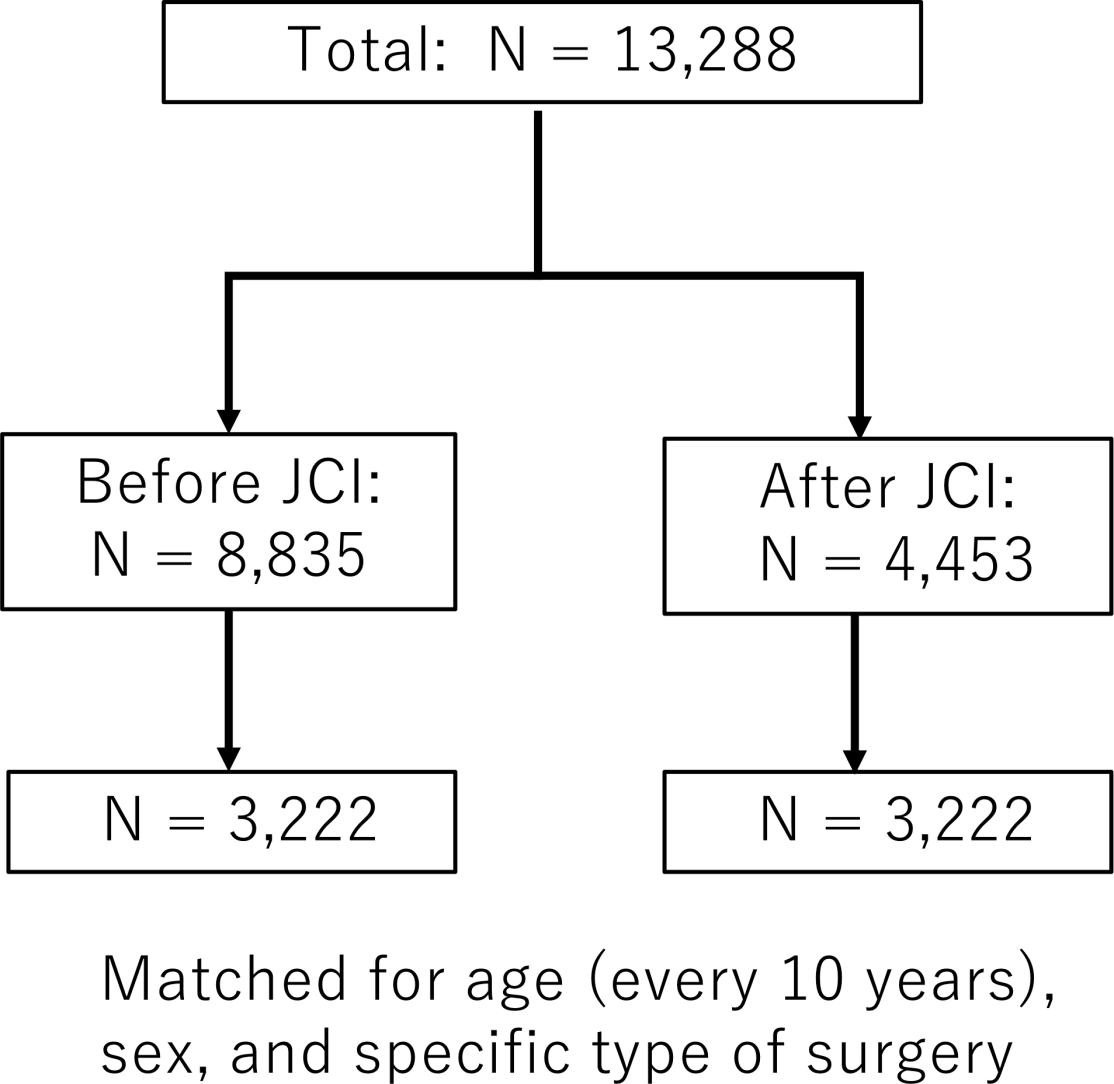
The flow diagram. A total of 13,288 patients were included in this study. Of the 8,835 and 4,453 patients receiving surgery before and after JCI accreditation, 3,222 pairs were matched for age (every 10 years), sex, and specific type of surgery. JCI = Joint Commission International.

Of the 8,835 and 4,453 patients receiving surgery before and after JCI accreditation, 3,222 pairs were matched for age, sex and specific surgery (Fig 2), resulting in groups for comparison (Tables 1 and 2). Table 3 shows the time periods in the operating room between matched patients before and after JCI accreditation. The TPT (197.4 ± 133.3 minutes vs. 195.2 ± 131.9 minutes, before vs. after JCI, p = 0.494) was not significantly different between groups. The preAT was significantly increased after JCI accreditation (8.2 ± 6.9 minutes vs. 8.5 ± 6.9 minutes, before vs. after JCI, respectively, p = 0.028), whereas the AIT was significantly reduced after JCI accreditation (34.4 ± 16.1 minutes vs. 33.6 ± 15.4 minutes, before vs. after JCI, respectively, p = 0.037). However, PT (42.6 ± 18.0 minutes vs. 42.2 ± 17.4 minutes, before vs. after JCI, p = 0.318) and postPT (20.7 ± 11.7 minutes vs. 20.6 ± 10.8 minutes, before vs. after JCI, p = 0.920) were not significantly different between groups.

**Table 3:** Time periods in the operating room between matched patients before and after JCI accreditation.

	Before JCI	After JCI	
Time periods, minutes (SD)	N = 3,222	N = 3,222	p value
Pre-anesthesia time	8.2 (6.9)	8.5 (6.9)	*0.028
Anesthesia induction time	34.4 (16.1)	33.6 (15.4)	*0.037
Pre-procedure/surgery time	42.6 (18.0)	42.2 (17.4)	0.318
Procedure/surgery time	134.1 (116.2)	132.3 (114.8)	0.534
Anesthesia awareness time	16.3 (10.9)	16.2 (10.2)	0.587
Post-anesthesia time	4.4 (5.1)	4.5 (4.8)	0.351
Post-procedure/surgery time	20.7 (11.7)	20.6 (10.8)	0.920
Total procedure/surgery time	197.4 (133.3)	195.2 (131.9)	0.494

JCI; Joint Commission International, SD; standard deviation. p values are calculated using the unpaired t-test (*<0.05).

### Subgroup analyses

AIT among patients who underwent total knee arthroplasty, and AAT and postPT among patients who underwent laparoscopic cholecystectomy were significantly decreased after JCI accreditation (Table 4A–4C). However, TPT was not significantly changed after JCI accreditation.

**Table 4:** The time periods in the operating room for three common and standardized surgeries before and after JCI accreditation.

**A. Total hip arthroplasty**			
	Before JCI	After JCI	
Time periods, minutes (SD)	n = 66	n = 66	p value
Pre-anesthesia time	6.3 (4.3)	6.8 (3.6)	0.456
Anesthesia induction time	38.5 (7.7)	38.9 (8.9)	0.770
Pre-procedure/surgery time	44.8 (7.4)	45.7 (9.4)	0.524
Procedure/surgery time	116.2 (35.3)	122.0 (36.1)	0.358
Anesthesia awareness time	20.0 (6.2)	19.0 (7.3)	0.390
Post-anesthesia time	5.3 (4.3)	5.5 (4.9)	0.778
Post-procedure/surgery time	25.4 (6.1)	24.6 (7.6)	0.514
Total procedure/surgery time	186.4 (39.2)	192.3 (33.8)	0.358
**B. Total knee arthroplasty**			
	Before JCI	After JCI	
Time periods, minutes (SD)	n = 41	n = 41	p value
Pre-anesthesia time	14.5 (7.4)	17.1 (8.2)	0.131
Anesthesia induction time	32.5 (7.2)	28.4 (8.5)	*0.020
Pre-procedure/surgery time	47.0 (9.1)	45.5 (7.8)	0.429
Procedure/surgery time	103.4 (20.7)	102.5 (28.2)	0.866
Anesthesia awareness time	16.3 (7.1)	15.7 (5.8)	0.697
Post-anesthesia time	5.6 (5.6)	4.4 (4.6)	0.233
Post-procedure/surgery time	21.9 (6.6)	20.1 (6.0)	0.184
Total procedure/surgery time	172.4 (26.1)	168.2 (30.9)	0.508
**C. Laparoscopic cholecystectomy**			
	Before JCI	After JCI	
Time periods, minutes (SD)	n = 37	n = 37	p value
Pre-anesthesia time	8.5 (6.3)	9.8 (6.5)	0.417
Anesthesia induction time	43.0 (7.6)	43.4 (9.5)	0.850
Pre-procedure/surgery time	51.5 (7.4)	53.1 (9.9)	0.435
Procedure/surgery time	136.9 (31.8)	138.6 (41.1)	0.848
Anesthesia awareness time	32.7 (10.8)	24.5 (10.6)	**0.002
Post-anesthesia time	4.9 (7.4)	4.6 (4.1)	0.818
Post-procedure/surgery time	37.6 (11.4)	29.1 (9.9)	**0.001
Total procedure/surgery time	226.0 (36.0)	220.8 (41.6)	0.562

JCI; Joint Commission International, SD; standard deviation. p values are calculated using the unpaired t-test (*p<0.05, **p<0.01).

## Discussion and conclusions

The demands of an aging society coupled with a progressively shrinking workforce have led to a financial crisis in trying to meet the needs of universal health care [5, 9]. Various measures, strategies and systems have been considered to curb the growth of medical expenses with only moderate effect [10]. Under such conditions, individual hospitals must strive to improve the efficiency of medical treatment with their limited income but without sacrificing the quality of medical care [2]. Hospitals should focus on the efficient management of surgical hospitalizations [11], as the operating room is an important source of revenue and accounts for more than 40% of hospital income [12]. Especially in acute care hospitals, the number of inpatients and outpatients is directly affected by operating room efficiency. In this study, we analyzed the time periods of standardized operating room procedures before and after JCI accreditation.

Hospital accreditation has become increasingly important to guarantee the quality of medical care and patient safety [4, 13]. JCI requires rigorous enforcement of IPSG to uphold quality medical care and promote continuous quality improvement of provided services. JUH has implemented surgical record sheets in their electronic medical records to ensure adherence to IPSG standards (S1 Table). Our data shows that preAT was significantly increased after JCI accreditation, possibly due to IPSG improvements in patient identification and medical staff communication. Despite that, the increased preAT was small, and the other time periods and TPT were not significantly affected, indicating that standardization of surgical parameters may reduce certain surgical time periods without significantly increasing TPT. The reduced AIT reflects a possibility that standardization of operating room procedures helps promote a clear understanding of each individuals responsibilities and enhances communication among team members [14], resulting in both improved patient safety and operating room efficiency [15].

In the subgroup analysis, the PTs were constant before and after JCI accreditation. Although preATs were also not significantly different, there was a slight increase in both groups before and after JCI accreditation, likely due to a lack of statistical power. TPT was unaffected in the subgroup analysis, supporting that implementation of standardized IPSG by JCI does not affect TPT.

Optimizing time periods in the operating room are important for the management and improvement of operating room resource utilization. Here, we revealed data on TPT and its five component time periods. These detailed data identify critical points in operation room management and help optimize planning, communication, and timing. Real-time implementation should help avoid bottle necks in operating room work flow. Ideally, the system can automatically track patients and resources and monitoring overall operating room performance [16]. Our system still requires manual input of information, but wireless patient tracking systems can timestamp key events more accurately [17].

There are several limitations to this study. First, data from a single university hospital may be difficult to generalize given inherent differences in hospital size, services provided, and patient population. However, it is reasonable to assume similar dynamics in hospitals worldwide, given the increasing trend of gaining external accreditation in hospitals. Second, we were not able to adjust for some factors that may have affected the operating time such as differences in the skills of the anesthesiologists and surgeons, differences in elective compared with emergent surgeries, and disease severity [18]. In addition, the accuracy of the data may be affected by manually recording the time intervals in the operating room. Third, despite our decision to dichotomize groups based on the date of JCI accreditation, IPSG measures may have affected clinical practice more gradually, resulting in a possible underestimation of the effects of IPSG on surgical time periods. In addition, S1 Fig shows the stable trend of incidents where further treatment is required after surgical intervention due to medical errors. Thus, the introduction of IPSG according to JCI had a small influence on the trend of incidents in the operating room, since the there is a few accidents rate in JUH. Finally, in our subgroup analysis, there were no significant differences in time periods between groups (except for AIT among patients who underwent total knee arthroplasty, and AAT and postPT among patients who underwent laparoscopic cholecystectomy), but this may be due to lack of statistical power.

In summary, here we investigated the impact of JCI accreditation and implementation of standardized procedures on time periods in the operating room. preAT was significantly increased, AIT was significantly reduced, and TPT was unchanged after implementing IPSG. Therefore, we concluded that patient safety and operating room efficiency can be compatible.

## Supporting information

S1 File Dataset. (XLS)

S2 File STROBE_checklist. (PDF)

S1 Table Steps for pre-anesthesia time and anesthesia induction time. (PDF)

S2 Table The distribution of the department before matching. (PDF)

S3 Table Time periods in the operating room before matching. (PDF)

S1 Fig The trend of incidents, which is when continuous treatment is required following surgery due to an incident. (PDF)
