# Supplementary material for: The impact of Joint Commission International accreditation on time periods in the operating room: A retrospective observational study
Source: PLoS One. 2018 Sep 21;13(9):e0204301. doi: 10.1371/journal.pone.0204301 (PMC6150533; doi:10.1371/journal.pone.0204301)
Supplement: S1 Table — (PDF) [file pone.0204301.s003.pdf]

S1 Table. Steps for pre-anesthesia time and anesthesia induction time.

	Steps for pre-anesthesia time (Sign in)
1	Identify the patient himself his / her name (full name) and birth of date
2	Surgical site and the operative side to the patient
3	Marking of surgical site
4	Allergies
5	Moving teeth, false teeth, a tooth under treatment
6	Restricted limbs, range of joint motion
7	A biological monitor is worn by the patient and is operating normally
8	Significant changes in vital signs before surgery

	Steps for anesthesia induction time (Time out)
1	All team members introduce their names and roles by themselves
Confirmation by physician	
2	Patient name, date of birth
3	Surgical method and surgical procedure
4	Confirmation of skin incision location and site
5	Important points of surgery
6	Scheduled operation time
7	Expected bleeding volume
8	Confirm installation of neutral zone
Confirmation by anesthesiologist	
9	Implementation of antimicrobial prophylaxis within 60 minutes before surgery
10	Blood transfusion preparation
11	Patient's specific problems
Confirmation by nursing team	
12	Sterilization of equipment and materials used for surgery
13	Problems to be shared within the team with allergies, equipment etc.
14	Display necessary images
15	Operation of intermittent pneumatic device
