# Supplementary material for: The impact of Joint Commission International accreditation on time periods in the operating room: A retrospective observational study
Source: PLoS One. 2018 Sep 21;13(9):e0204301. doi: 10.1371/journal.pone.0204301 (PMC6150533; doi:10.1371/journal.pone.0204301)
Supplement: S2 Table — (PDF) [file pone.0204301.s004.pdf]

S2 Table. **The distribution of the department before matching.**

Department	Before JCI	After JCI	p value
Breast oncology	393	206	0.075
Cardiovascular surgery	659	312	
Coloproctological surgery	383	177	
Esophageal and Gastroenterological surgery	320	150	
Gynecology	1,113	533	
Hepatobiliary-Pancreatic surgery	320	174	
Neurosurgery	489	301	
Obstetrics	341	200	
Ophthalmology	306	174	
Orthopedics	848	453	
Otolaryngology	706	332	
Pediatric surgery	1,205	606	
Plastic surgery	474	221	
Thoracic surgery	533	239	
Urology	646	331	
Others	99	44	
Total	8,835	4,453	

JCI; Joint Commission International, p value is calculated by chi-square test.
