# Supplementary material for: The impact of Joint Commission International accreditation on time periods in the operating room: A retrospective observational study
Source: PLoS One. 2018 Sep 21;13(9):e0204301. doi: 10.1371/journal.pone.0204301 (PMC6150533; doi:10.1371/journal.pone.0204301)
Supplement: S3 Table — (PDF) [file pone.0204301.s005.pdf]

S3 Table. **Time periods in the operating room before matching.**

Time periods, minutes (SD)	Before JCI n = 8,835	After JCI n = 4,453	p value
Pre-anesthesia time	8.3 (7.0)	8.5 (6.9)	0.056
Anesthesia induction time	35.6 (16.7)	34.8 (16.4)	*0.010
Pre-procedure/surgery time	43.9 (18.5)	43.3 (18.3)	0.108
Procedure/surgery time	139.8 (124.7)	136.3 (124.8)	0.126
Anesthesia awareness time	16.7 (10.6)	16.9 (10.4)	0.420
Post-anesthesia time	4.5 (5.6)	4.5 (4.7)	0.914
Post-procedure/surgery time	21.2 (11.5)	21.4 (11.0)	0.424
Total procedure/surgery time	204.9 (141.6)	201.0 (142.1)	0.136

JCI; Joint Commission International, SD; standard deviation. p values are calculated using the unpaired t-test (*$p<0.05$).
