# Supplementary material for: The impact of Joint Commission International accreditation on time periods in the operating room: A retrospective observational study
Source: PLoS One. 2018 Sep 21;13(9):e0204301. doi: 10.1371/journal.pone.0204301 (PMC6150533; doi:10.1371/journal.pone.0204301)
Supplement: S1 Fig — (PDF) [file pone.0204301.s006.pdf]

S1 Fig. The trend of incidents, which is when continuous treatment is required following surgery due to an incident.

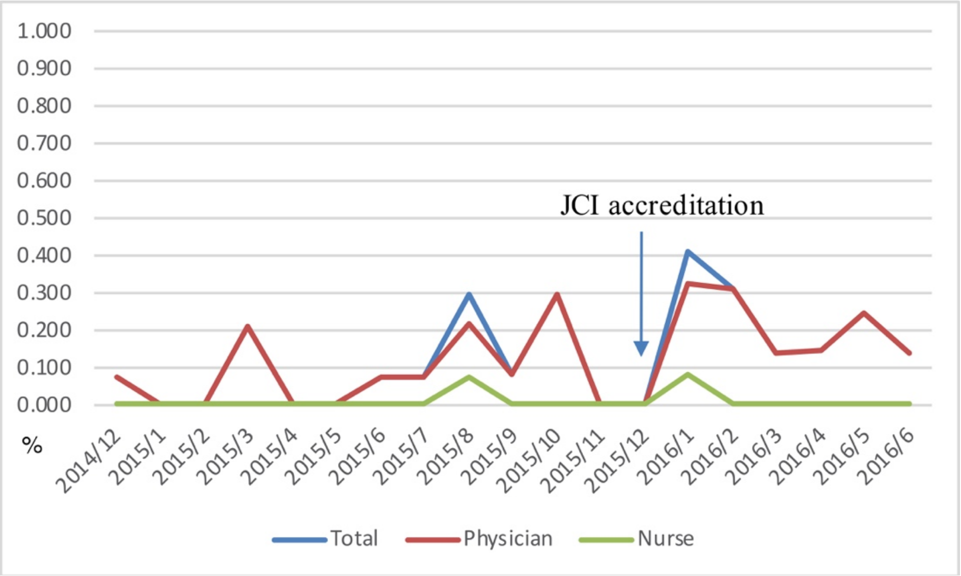

JCI; Joint Commission International.
